# Optimization of Agroinfiltration in *Pisum sativum* Provides a New Tool for Studying the Salivary Protein Functions in the Pea Aphid Complex

**DOI:** 10.3389/fpls.2016.01171

**Published:** 2016-08-09

**Authors:** Endrick Guy, Hélène Boulain, Yoann Aigu, Charlotte Le Pennec, Khaoula Chawki, Stéphanie Morlière, Kristina Schädel, Grit Kunert, Jean-Christophe Simon, Akiko Sugio

**Affiliations:** ^1^INRA, UMR1349, Institute of Genetics, Environment and Plant ProtectionLe Rheu, France; ^2^Department of Biochemistry, Max Planck Institute for Chemical EcologyJena, Germany

**Keywords:** pea aphid, *Acyrthosiphon pisum*, Leguminosae, agroinfiltration, salivary proteins, biotypes, host specialization, effector

## Abstract

Aphids are piercing-sucking insect pests and feed on phloem sap. During feeding, aphids inject a battery of salivary proteins into host plant. Some of these proteins function like effectors of microbial pathogens and influence the outcome of plant–aphid interactions. The pea aphid (*Acyrthosiphon pisum*) is the model aphid and encompasses multiple biotypes each specialized to one or a few legume species, providing an opportunity to investigate the underlying mechanisms of the compatibility between plants and aphid biotypes. We aim to identify the aphid factors that determine the compatibility with host plants, hence involved in the host plant specialization process, and hypothesize that salivary proteins are one of those factors. *Agrobacterium*-mediated transient gene expression is a powerful tool to perform functional analyses of effector (salivary) proteins in plants. However, the tool was not established for the legume species that *A. pisum* feeds on. Thus, we decided to optimize the method for legume plants to facilitate the functional analyses of *A. pisum* salivary proteins. We screened a range of cultivars of pea (*Pisum sativum*) and alfalfa (*Medicago sativa*). None of the *M. sativa* cultivars was suitable for agroinfiltration under the tested conditions; however, we established a protocol for efficient transient gene expression in two cultivars of *P. sativum*, ZP1109 and ZP1130, using *A. tumefaciens* AGL-1 strain and the pEAQ-HT-DEST1 vector. We confirmed that the genes are expressed from 3 to 10 days post-infiltration and that aphid lines of the pea adapted biotype fed and reproduced on these two cultivars while lines of alfalfa and clover biotypes did not. Thus, the pea biotype recognizes these two cultivars as typical pea plants. By using a combination of ZP1109 and an *A. pisum* line, we defined an agroinfiltration procedure to examine the effect of *in planta* expression of selected salivary proteins on *A. pisum* fitness and demonstrated that transient expression of one candidate salivary gene increased the fecundity of the aphids. This result confirms that the agroinfiltration can be used to perform functional analyses of salivary proteins in *P. sativum* and consequently to study the molecular mechanisms underlying host specialization in the pea aphid complex.

## Introduction

Herbivorous insects present a high level of species diversity and a large majority of them is specialized to feed on certain host plant species. Specialization to different host plants also occurs within single insect species and leads to the existence of distinguishable “host races” or “biotypes” (Dres and Mallet, 2002). The mechanisms of host plant adaptation in herbivorous insects are poorly understood, although these could explain a large part of insect species richness (Simon et al., 2015). Therefore, insect species displaying an array of races or biotypes provide interesting opportunities to study the process of host plant specialization due to the possibility to compare genomes and feeding strategies between closely related races or biotypes.

The pea aphid, *Acyrthosiphon pisum* Harris, is the first aphid to be genome sequenced and owing to its long history of research, it is the model of aphids and sap-feeding insects (hemipterans; International Aphid Genomics Consortium, 2010). In addition, *A. pisum* encompasses a range of biotypes each specialized to one or a few closely related legume species but cannot survive or reproduce well on non-host legume plants. So far, 15 biotypes are described (Peccoud et al., 2015), of which alfalfa, clover and pea biotypes are the ones most studied in host specialization (Hawthorne and Via, 2001; Ferrari et al., 2008; Peccoud et al., 2009; Jaquiery et al., 2012; Via et al., 2012). In addition to show strong differences in performances on host and non-host plants, these biotypes are genetically distinct and can be distinguished by using microsatellite markers (Ferrari et al., 2008; Peccoud et al., 2009). Interestingly, all the *A. pisum* biotypes studied so far feed well on *Vicia faba*, which is considered as a universal host plant for pea aphids (Ferrari et al., 2008; Peccoud et al., 2009). Many of these *A. pisum* biotypes can be crossed with other biotypes (Peccoud et al., 2014), and QTL analyses have been used to identify aphid factors that determine the compatibility with the host plants (Hawthorne and Via, 2001; Via et al., 2012; Kanvil et al., 2015).

Aphids feed on plant phloem sap using a specialized mouthpart called stylet. During feeding, aphids may transmit plant pathogenic viruses, inject toxic saliva and remove nutrients from host plants. Hence, aphids are considered among the most serious crop pests. Recent studies gradually revealed that there are intricate molecular interactions between the proteins secreted with aphid saliva and host plant proteins (Elzinga and Jander, 2013; Rodriguez and Bos, 2013; Kaloshian and Walling, 2016). In some cases, salivary proteins trigger plant defense responses (De Vos and Jander, 2009; Chaudhary et al., 2014; Elzinga et al., 2014), in others, they suppress plant defense reactions and promote aphid proliferation (Will et al., 2007; Bos et al., 2010; Atamian et al., 2013; Elzinga et al., 2014; Naessens et al., 2015). Hence, aphid salivary proteins are considered to be analogous to effectors of plant pathogens, and their functions have been examined using similar techniques, such as silencing of salivary genes or *in planta* expression of salivary proteins (Elzinga and Jander, 2013; Rodriguez and Bos, 2013). The first characterized aphid salivary gene was an *A. pisum* gene named C002, which is strongly expressed in salivary glands and was detected in plants infested by the aphids. Silencing of *A. pisum C002* (*ApC002*) was achieved by injection of siRNA in aphids. It prevented aphids from feeding on *V. faba*, while aphid feeding on artificial diet was unaffected (Mutti et al., 2006, 2008). In line with these studies, transient or stable expression of *Myzus persicae* orthologue of ApC002, MpC002, in *Nicotiana benthamiana* and *Arabidopsis thaliana*, respectively, increased the fecundity of *M. persicae* feeding on these plants, indicating the conserved role of C002 as an effector required for aphid feeding on host plants (Bos et al., 2010; Pitino and Hogenhout, 2013; Elzinga et al., 2014).

Since then, several *A. pisum* salivary proteins required for aphid full performance have been identified and characterized mostly by using gene silencing induced by siRNA injection to aphids (Guo et al., 2014; Pan et al., 2015; Wang et al., 2015a,b) while several salivary proteins from other aphids, such as *M. persicae* have been identified using transient or stable *in planta* expression of salivary genes (Bos et al., 2010; Pitino and Hogenhout, 2013; Elzinga et al., 2014). However, since the *A. pisum* genome is extensively duplicated and more than 2000 gene families show massive expansion compared to published insect genomes (Rispe et al., 2008; International Aphid Genomics Consortium, 2010; Jaubert-Possamai et al., 2010), it is often difficult to select a siRNA or dsRNA fragment that specifically targets the gene of interest for silencing. In some cases, co-silencing of multiple gene family members need to be examined to determine whether the phenotype observed is due to the silencing of single gene or multiple genes. Furthermore, there is a possibility that gene silencing does not show a strong phenotypic effect on plant–aphid interactions if genes with redundant functions exist or if gene silencing is too transient.

On the other hand, *in planta* expression of saliva gene allows simple characterization of single gene in plant-aphid interactions. While the construction and multiplication of transgenic plants require several months to years of preparation before testing, *Agrobacterium* mediated transient gene expression (agroinfiltration) can be achieved in a few days; therefore, it is a commonly used technique to identify and characterize effector functions. However, the efficiency of agroinfiltration is highly variable and often depends on the compatibility between the *Agrobacterium tumefaciens* strain and the plant species or cultivar used (Wroblewski et al., 2005). The technique has been developed in *N. benthamiana* using a disarmed strain where the virulence factors encoded by the Ti plasmid were deleted (Goodin et al., 2008). Then, the technique was optimized for different plants such as potato (Bhaskar et al., 2009), lettuce (Chen et al., 2016), grapevine (Santos-Rosa et al., 2008), *Medicago truncatula* (Picard et al., 2013) and recently in soybeans (King et al., 2015). However, the technique is not established in the legume plants, which are hosts for *A. pisum*.

As mentioned earlier, *A. pisum* encompasses multiple biotypes which cannot survive on the plants they are not specialized to. We study the commonest and most studied pea aphid biotypes to identify the factors that determine the compatibility between the aphid and legume species as such factors are likely be involved in the host plant specialization process of the aphids. Based on our recent genome analysis of three aphid biotypes respectively specialized on clover, alfalfa and pea, we hypothesized that salivary proteins are one of the factors that are involved in the host plant specialization process in *A. pisum* (Jaquiery et al., 2012). Hence, we envisaged to identify salivary proteins with biotype specific polymorphisms and to characterize their effects on specific plant–aphid interactions. Some salivary proteins from non-adapted biotypes may induce resistance responses in non-host plants while some salivary proteins from adapted biotypes may suppress specific plant defense reactions and allow non-adapted aphids to feed on non-host plants.

Here, as the first step to reach the objectives and to facilitate identification and functional characterization of *A. pisum* salivary proteins, we undertook optimization of agroinfiltration in *Medicago sativa* (alfalfa) and *Pisum sativum* (pea). We focused on these two plants because (1) significant amount of studies have been done on the aphid biotypes that feed on these plants (Hawthorne and Via, 2001; Jaquiery et al., 2012; Via et al., 2012), (2) these two biotypes show clear-cut performance difference on these two plants (Peccoud et al., 2009), and (3) seeds of various cultivars are easily available in our research center.

## Materials and Methods

### Aphids, Bacteria Strains, Plasmids and Growth Conditions

Aphid lineages, and bacterial strains and plasmids used in this study are listed in Supplementary Tables S1 and S2, respectively. All aphid lineages were reared in a growth chamber at 18°C with a 16 h day/8 h night photoperiod on the broad bean, *Vicia faba* (Castel), at low density to avoid the production of winged individuals. *Escherichia coli* and *A. tumefaciens* strains were grown on Luria-Bertani medium at 37°C and 30°C, respectively. For solid media, agar was added at a final concentration of 1.5% (w/v). Antibiotics were used at the following concentrations: for all bacteria, 50 μg/ml kanamycin; for *A. tumefaciens*, 50 μg/ml rifampicin; for *E. coli*, 10 μg/ml gentamycin.

### Plants and Growth Conditions

*Pisum sativum* (Supplementary Table S3) and *Medicago sativa* plants were grown in a growth chamber at 18°C with a 16 h day/8 h night photoperiod for 2 and 3 weeks, respectively.

### Measurements of Aphid Performances on Pea Cultivars

Life traits of five aphid lineages from pea (Ar_Po_28, Ar_Po_58), alfalfa (L9Ms14) and clover (YR2, T8005) biotypes (Supplementary Table S1) were measured on *P. sativum* cultivars ZP1130 and ZP1109 (Supplementary Table S3). Adult aphids were installed on both pea cultivars and removed 24 h later, giving them enough time to produce 10 larvae that were left on the plants (day 1). Survival rate of the 10 larvae was measured at day 9 (when they reach adulthood), three surviving adult aphids were then reinstalled on the plants and biomass (the cumulated weight of the three adults and their offspring) of the aphid population was weighted at day 17. The biomass is a good proxy of the number of nymphs produced by adult aphids and reflects well their overall fitness (Peccoud et al., 2009). Five replicates for each aphid lineage on the two tested plants were performed.

### Construction of Plasmids

All primers used in this study are listed in Supplementary Table S4. The genes encoding eGFP and the β-glucuronidase with a plant derived intron (GUSi; Vancanneyt et al., 1990) were amplified using GFP-Fw/GFP-Rv primers and GUS-Fw/GUS-Rv primers, respectively, and were added complete *attB1* and *attB2* sequences by the second PCR with attB1 and attB2 primers. In order to clone aphid salivary genes, cDNAs produced from aphid head total RNA were used to enrich transcripts encoding salivary genes. Adult aphids feeding on *V. faba* were flash frozen in liquid nitrogen, and decapitated with a scalpel between the first and second pairs of legs. Head RNA was extracted from 10 to 20 individuals using the RNeasy plant mini kit (Qiagen). cDNA synthesis was performed with poly-T primers using the AMV reverse transcriptase system (Promega) according to the manufacturers’ instructions. ACYPI009919 (Ap25) and ACYPI008617 (ApC002) open reading frames encoding mature proteins were amplified from the cDNA of the Ar_Po_58 line (pea biotype) with Phusion DNA polymerase (ThermoFisher Scientific) using AP25-Fw/AP25-Rv and APC002-Fw/APC002-Rv primers (Supplementary Table S4), respectively. *attB1* and *attB2* sites were added with a second PCR using attB1 and attB2 primers. All amplicons, eGFP, GUSi and two salivary genes, were recombined by BP reaction into pDONR207 (Invitrogen) using BP clonase II (Invitrogen) and produced entry vectors (Table S2). Entry vectors were recombined by LR reaction using LR clonase II (Invitrogen) into pEAQ-HT-DEST1 expression vector (Supplementary Table S2; Sainsbury et al., 2009). Expression vectors were transformed in electro-competent *A. tumefaciens* cells (Supplementary Table S2).

### Infiltration of *Agrobacterium*

*Agrobacterium tumefaciens*-mediated transient expression was performed as described (Rivas et al., 2002). Freshly cultured cells were resuspended in induction buffer [10 mM MgCl_2_, 10 mM Mes (2-(*N*-morpholino) ethanesulfonic acid), pH 5.6, and 150 μM acetosyringone] to an O.D._600_ (optical density at 600 nm) of 0.5. Cells were syringe infiltrated into leaves of 2 week-old *P. sativum* (Supplementary Table S1) and 3 week-old *M. sativa* plants.

### GUS Staining

Plant leaves infiltrated with *Agrobacterium* were detached 3 days post infiltration (dpi) and GUS activity was visualized as described (Jefferson, 1987). Briefly, leaves were vacuum infiltrated with GUS staining solution (61 mM Na_2_HPO_4_, 39 mM NaHPO_4_, 0.1% triton X-100, 10 mM EDTA, 0.3% H_2_O_2_ and 1.5 mM 5-bromo, 4-chloro, 3-indolyl glucuronide (X-Glc, Biosynth), pH 7.0) and incubated overnight at 37°C. Then chlorophyll discoloration was performed with successive washes with ethanol at 37°C.

### Protein Extraction and Western-Blot Analyses

Three leaf disks per leaf were sampled using a cork borer (area = 0.79 cm^2^) at 0, 7, and 10 days post-infiltration for GFP protein detection. Leaf disks were flash frozen in liquid nitrogen and stored at -80°C. Proteins were extracted in 120 μl extraction buffer (50 mM tris pH 7.5, 1 μM Dithiothreitol, glycerol 10%, 1 mM PMSF (Phenylmethylsulfonyl fluoride), 0.05% triton X-100). Extracts from pea plants were prepared as described (Canonne et al., 2011) and supernatants were resuspended in 5X loading buffer (0.5 M Tris pH 6.8, SDS 10%, glycerol 50 and 0.001% bromophenol blue). Fifteen microliters of samples were separated by SDS-PAGE (12% polyacrylamide) and transferred on PVDF (Polyvinylidene fluoride) membranes, (Merck Millipore) as described (Witte et al., 2004) with following modifications: PVDF membranes were soaked in methanol before and after transfer, and then washed in water. Methanol in transfer buffer was replaced by ethanol. The rabbit anti-GFP antibody (Biorad) and secondary antibodies (polyclonal goat anti-rabbit antibody peroxidase conjugated; Sigma–Aldrich) were both used at 1:10000. Detection was performed by chemiluminescence using Clarity Western ECL Substrate (Biorad) and CL-XPosure^TM^ Film (Lifetechnologies) according to manufacturer’s instructions. Coomassie stains were performed with 0.2% Coomassie Brilliant Blue R250 (Sigma) in 50:40:10 water, methanol, acetic acid.

### Aphid Performance Test on Agroinfiltrated Leaves

One young leaf of the *P. sativum* ZP1109 cultivar was syringe-infiltrated with *A. tumefaciens* AGL-1 strain harboring expression vectors. Three days later (at 3 dpi), 6 new-born aphids (1 day-old) born on *V. faba* were installed on *P. sativum* agroinfiltrated leaves in custom-built clip cages (area = 2.54 cm^2^). When aphids were 8 days-old (10 dpi), clip cages were opened and the number of surviving aphids was recorded to estimate the survival rate. From the survivors, one average sized aphid was selected and transferred to a new *P. sativum* leaf that was infiltrated with the same construct of *Agrobacterium* 3 days before the transfer. Clip cages were opened when aphids were 12 and 15 days-old to assess the fecundity by counting the number of nymphs produced by each aphid. The nymphs were removed after each counting to avoid overcrowding of the cages. In one experiment, 10 replicates per gene were performed and the same experiment was repeated twice, producing 20 replicates. All the experiments were conducted at 20°C, 16 h day/8 h night photoperiod.

### Statistical Analyses

All statistical analyses were conducted in R version 3.1.2 (R Core Team, 2014). Data were checked for approximate normal distribution by graphical visualizing of residuals. The effects of the different factors (pea cultivar, aphid lineage, expressed gene) were tested and the simplest model explaining the data was used. Analyses of survival rates (**Figures 2A** and **3A**) and fecundity counts (**Figure 3B**) were performed by classical linear regressions using generalized linear models (GLM) with binomial and Poisson distributions, respectively. Both tests were followed by multiple comparisons of means by the Tukey contrast method implemented in the package “multcomp” (Hothorn et al., 2008). The influence of pea cultivars and aphid lineage on aphid biomass (**Figure 2B**) was analyzed by a two-way ANOVA. Tukey’s *post hoc* multiple comparisons of means from the R package “agricolae” (De Mendiburu, 2014) were used to reveal differences between groups.

## Results

### Screening of *P. sativum* and *M. sativa* Cultivars for Agroinfiltration

**FIGURE 1:**
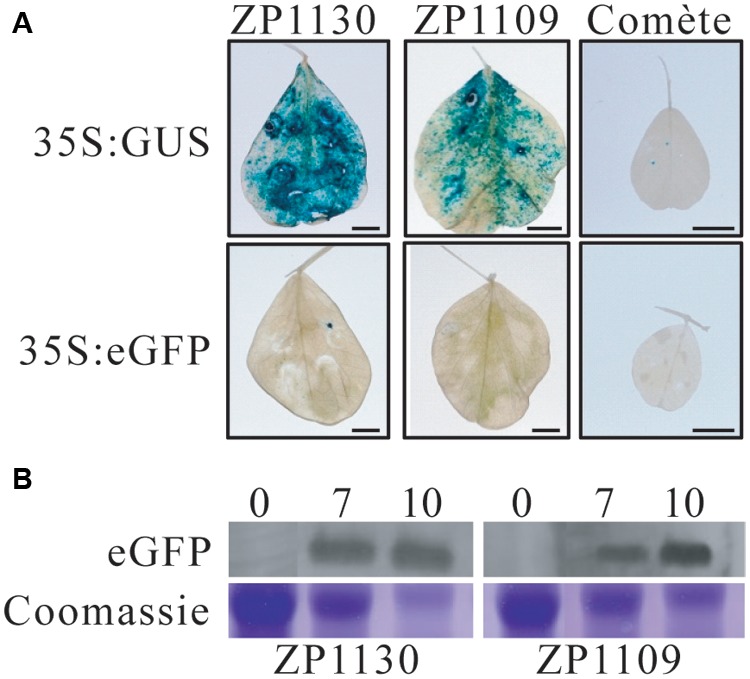
**Optimization of agroinfiltration in legume plants. (A)** GUS expression in *P. sativum* and *M. sativa*. Leaves of ZP1130 and ZP1109 cultivars of *P. sativum* and comète cultivar of *M. sativa* were syringe-infiltrated with *A. tumefaciens* AGL-1 carrying the pEAQ-HT-DEST1 plasmid encoding the gene for β-glucuronidase with an intron (GUSi), under the control of the CaMV 35S promoter (35S:GUSi). GUS staining was performed 3 days post infiltration. Results are representatives of four independent experiments. *A. tumefaciens* carrying the pEAQ-HT-DEST1 plasmid encoding the gene for enhanced GFP, under the control of the CaMV 35S promoter (35S:eGFP) was used as control. Scale bars represent 0.5 cm. **(B)** Total protein extracts of leaves from ZP1130 and ZP1109 cultivars expressing eGFP using *Agrobacterium*-mediated transformation were separated by 12% SDS-PAGE and analyzed by immunoblot using the anti-GFP antibody. Samples were harvested at 0, 7 and 10 days post infiltration. Coomassie stained portions of the gel (Rubisco) are shown to compare sample loading between lanes.

Combinations of *A. tumefaciens* and various *M. sativa* and *P. sativum* cultivars were tested using the β-glucuronidase containing a plant derived intron (GUSi) as a reporter gene (Vancanneyt et al., 1990). Green fluorescence protein (GFP) could not be used as a reporter due to strong auto fluorescence induced in the leaf surface by the infiltration. Initially, we tested two plant expression vectors pGWB402Ω (Nakagawa et al., 2007) and pEAQ-HT-DEST1 (Sainsbury et al., 2009) in some pea cultivars, but the difference in expression levels between the two vectors was not very clear or slightly better when pEAQ-HT-DEST1 was used. Therefore, we used pEAQ-HT-DEST1 for the rest of screening. Also, our initial test showed that a bacterial suspension with an O.D._600_ less than 0.3 resulted in a weak transgene expression and more than 0.7 triggered leaf chlorosis a few days after infiltration. Therefore, for the rest of the screening, agroinfiltrations were performed using syringe infiltration method and a bacterial suspension with an O.D._600_ = 0.5. Seventeen *P. sativum* (Supplementary Table S3) and five *M. sativa* cultivars were selected based on geographic origin and phylogenetic groups in order to screen a large genetic diversity. Each cultivar was infiltrated with three *Agrobacterium* strains [C58C1, GV3101 and AGL-1 (Supplementary Table S2)] each harboring pEAQ-HT-DEST1-GUSi to identify the combination of plant and bacterium genotypes that produce high amount of GUS proteins. Leaves were analyzed histochemically for GUS activity at 3 dpi. At least three independent experiments were performed for each combination and results are summarized in **Table 1**. None of the *M. sativa* cultivars was suitable for *Agrobacterium*-mediated transient expression in leaves as no GUS staining could be observed in these plants under the tested conditions. High differences between pea cultivars were observed. Most of the pea cultivars had no or weak intensities of GUS staining. Of the three *Agrobacterium* strains used in this study, AGL-1 induced the highest expression of GUS, and C58C1 was the lowest inducer. Two pea genotypes, ZP1130 and ZP1109, inoculated with AGL-1 showed most intense coloration during GUS staining (**Figure 1A**). GUS staining could be observed at 3 dpi for both cultivars, ZP1130 and ZP1109. To confirm protein expression in these two cultivars, transient expression of eGFP and detection by western-blot was performed (we could not visualize GFP fluorescence due to autofluorescence induced by wounding). eGFP protein was detected at 7 and 10 dpi for both ZP1109 and ZP1130 (**Figure 1B**). During this study, yellowing of the leaves starting at 9–10 dpi for ZP1130 and at 12–13 dpi for ZP1109 was observed. This leaf yellowing was probably due to AGL-1 infection as the yellowing was observed in the leaves infiltrated with *Agrobacterium* with empty vector control, and no yellowing was observed in buffer infiltrated leaves (data not shown). Taken together, we identified two pea cultivars, ZP1130 and ZP1109, and the *A. tumefaciens* strain AGL-1 as the combinations that are suitable for transient gene expression, and we presumed that 3–8 dpi for ZP1130 and 3–10 dpi for ZP1109 are the timing to examine the effect of transgene expression in the plant or plant–aphid interactions.

**Table 1:** Results of screening of *P. sativum* and *M. sativa* cultivars for agroinfiltration.

	C58C1^b^	GV301	AGL-1
***Pisum sativum***^a^			
AP3783	N	I	W
AP3830	N	N	N
WP1018	W	W	W
ZP690	N	N	N
ZP748	N	N	N
ZP750	N	W	W
ZP793	N	W	W
ZP747	N	N	W
ZP1109	N	I	I
ZP1124	N	N	W
ZP1130	N	W	S
ZP3495	N	W	N
ZP3508	N	N	N
ZP3514	N	N	N
ZP3535	N	N	N
ZP3570	N	N	N
ZP3664	N	W	W
***Medicago sativum***			
Comète	nd	nd	N
Harpe	nd	nd	N
Lux Timbale	nd	nd	N
Lux Galaxie	nd	nd	N
Cannelle	nd	nd	N

*^a^pea or alfalfa cultivars used in this study. ^b^*A. tumefaciens* strains used in this study. N, no coloration; W, weak; I, intermediate coloration; S, strong coloration (**Figure 1A**), nd, not determined.*

### Pea Cultivars ZP1130 and ZP1109 Are Hosts Only for the *A. pisum* Pea Biotype

**FIGURE 2:**
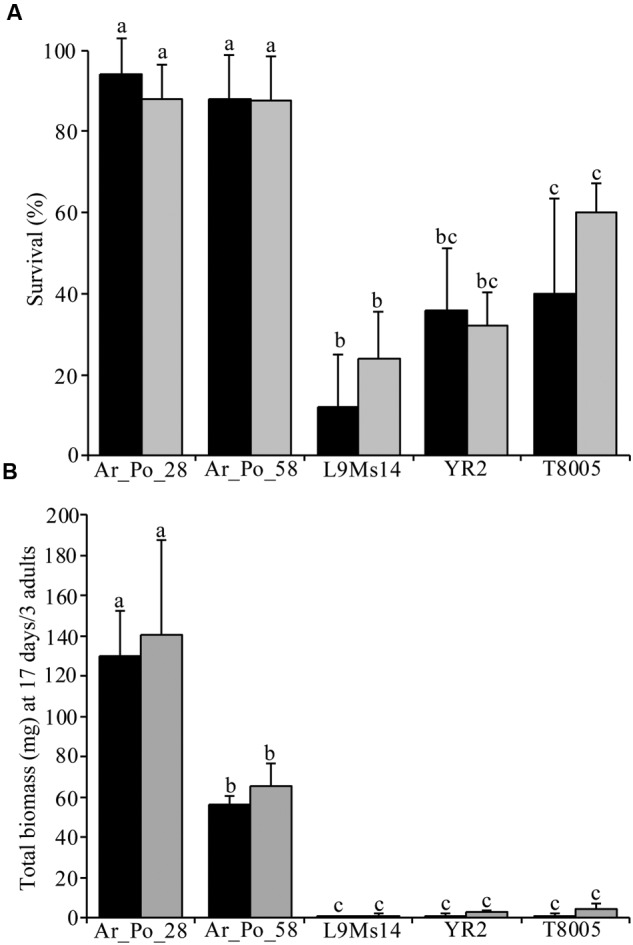
**ZP1109 and ZP1130 allow only *A. pisum* pea biotype reproduction.** Survival **(A)** and biomass **(B)** of five aphid lineages are measured on the pea cultivars ZP1109 (black bars) and ZP1130 (gray bars). Bars show the average of survival or biomass and standard deviation for five replicates per conditions. Statistical differences between groups are indicated by different letters. a, b, c; indicate groups determined by multiple comparisons tests after GLM and ANOVA analyses for survival and biomass data, respectively.

Survival rate and biomass of the five *A. pisum* lineages belonging to three biotypes (pea, alfalfa and clover; Supplementary Table S1) were assessed on the ZP1130 and ZP1109 pea cultivars we identified as suitable for agroinfiltration (**Figure 2**). Analysis revealed that the two plant cultivars did not influence the survival rate and produced aphid biomass [χ^2^*=* 0.14, *P =* 0.243; *F*_(5,44)_ *=* 129.7, *P =* 0.261; for survival and biomass, respectively], but pea aphid lineages differed significantly in their survival rates (χ^2^*=* 19.04, *P* < 0.001) and biomass production [*F*_(4,45)_*=* 128.9, *P* < 0.001]. The pea adapted lineages Ar_Po_28 and Ar_Po_58 showed a higher survival rate on the pea cultivars at day 9 compared to L9Ms14 (alfalfa biotype), YR2 and T8005 (clover biotype). The difference in survival was very pronounced between pea and alfalfa specialized lineages, and intermediate for lineages of the clover biotype (**Figure 2A**). On both ZP1130 and ZP1109 cultivars, only the lineages of the pea biotype (Ar_Po_28 and Ar_Po_58) produced a substantial biomass. Although Ar_Po_28 had a significantly higher biomass than Ar_Po_58, both lineages performed well on the tested cultivars that they seem to recognize as favorable hosts. By contrast, alfalfa and clover adapted lineages hardly reproduced on the pea cultivars that seem to be non-host plants in these interactions (**Figure 2B**). Thus, the ZP1130 and ZP1109 cultivars are selective hosts for *A. pisum* biotypes, allowing to assess host and non-host interactions using agroinfiltration experiments.

### Transient Expression of AP25 in ZP1109 Increased *A. pisum* Fecundity

Next, we expressed two salivary genes in ZP1109 by agroinfiltration using strain AGL-1 and examined their effects on *A. pisum* feeding on the infiltration site. We chose Ar_Po_58 as a test aphid line as it belongs to the pea biotype and harbors no secondary symbiont, which may interfere with plant–aphid interactions. Mature proteins encoding ACYPI008617 (ApC002) and ACYPI009919, which we named Ap25, were transiently expressed using pEAQ-HT-DEST1 vector. The genes were expressed by CaMV 35S promoter, which is known to be ubiquitously and constitutively activated in various plant tissues including epidermal, mesophyll and phloem tissues (Stockhaus et al., 1989). In the process of establishing phloem feeding, *A. pisum* punctures various tissues and salivates (Schwarzkopf et al., 2013). When the aphid attempts to feed on non-host legume plant, it punctures epidermal and mesophyll cells but cannot establish phloem feeding: therefore, the factors that determine the compatibility between *A. pisum* and host plants are present in those tissues (Schwarzkopf et al., 2013). Based on these informations, we thought it is important to express salivary proteins ubiquitously to fully assess their functions in plants and used 35S promoter for transient expression. 35S promoter has been successfully used in other studies on aphid salivary proteins (Bos et al., 2010; Naessens et al., 2015). *ApC002* was chosen because it is one of the most studied salivary proteins and is shown to be essential for *A. pisum* to feed on the universal host plant (*V. faba*; Ferrari et al., 2008; Peccoud et al., 2009). *Ap25* was selected because the gene presents the same features as that of *ApC002*: the gene was identified in salivary glands by transcriptomic analyses (Carolan et al., 2011), is specifically expressed in salivary glands (Akiko Sugio et al., unpublished data), and encodes a signal peptide and a small (13.9 kDa) mature protein with no predicted function. Although many genes are duplicated in *A. pisum* genome, *Ap25*, like *ApC002*, is single copy in *A. pisum* and its orthologues exist only in the Aphididae family (Hélène Boulain et al., unpublished data).

**FIGURE 3:**
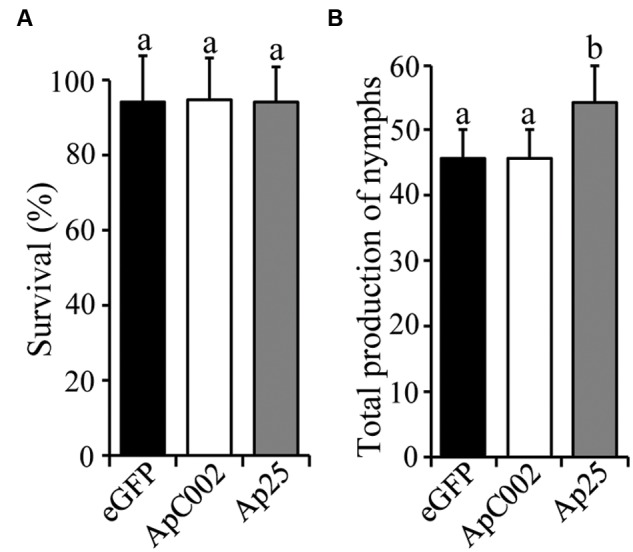
**Transient expression of Ap25 promotes reproduction of *A. pisum* on ZP1109.** Leaves of ZP1109 cultivar of *P. sativum* were syringe-infiltrated with *A. tumefaciens* AGL-1 carrying the pEAQ-HT-DEST1 plasmid encoding the genes for *ACYPI008617* (*ApC002*; white bar) and *ACYPI009919* (*Ap25*; gray bar), under the control of the CaMV 35S promoter. *A. tumefaciens* carrying the pEAQ-HT-DEST1 plasmid encoding the gene for enhanced GFP (eGFP; black bar), under the control of the CaMV 35S promoter was used as control. **(A)** Survival rate of Ar_Po_58 line is not affected by the transient expression of the salivary genes *ApC002* and *Ap25*. At 3 days post infiltration, six new-born aphids were clip caged on agroinfiltrated leaves and counted when aphids were adults (8 days-old) to check the survival rate. Bars show the average percentage of survivors plus the standard deviation of 20 biological replicates. After the survival test, one aphid per plant was kept and placed on a new 3-day-post-infiltrated leaf to check the fecundity. **(B)** The transient expression of different genes influenced aphid nymph production. Bars show the average number of nymphs produced plus the standard deviation of 20 biological replicates. Different letters indicate significant differences between groups.

In this study, transient protein expression was observed from 3 (detected by GUS activity) to 10 days (detected by western blot) at 20°C after infiltration of *Agrobacterium*. *A. pisum* starts to reproduce around 9th day after birth, reaches its peak of reproduction around 5 days later, and slows down but continues to reproduce until its death at an age of approximately 30 days (Tsuchida et al., 2004). By supplying newly infiltrated leaves, we extended the duration of the experiment to characterize the effect of transgene expression on aphid fecundity. Leaves of ZP1109 were infiltrated with AGL-1 harboring expression plasmids of eGFP, ApC002 or Ap25. Three days after the infiltration, six new-born aphids of the pea adapted clone Ar_Po_58 were clip caged on the infiltrated leaves. When the aphids were 8 days-old (at 10 dpi) the cages were opened to count the number of survivors. One aphid was transferred to a new 3-day-post-infiltrated leaf. Production of nymphs of the caged adult was measured when the aphid was 12 and 15-day old corresponding to the peak of reproduction of adults. Survival rate and total number of nymphs of the aphids are shown in **Figure 3**. There was no difference in the survival rate of the aphids that were fed on the leaves expressing the three tested genes (χ^2^*=* 0.01, *P* = 0.96). Production of nymphs of Ar_Po_58 feeding on ApC002 expressing leaves was same as that of aphids feeding on eGFP expressing leaves, while the aphids produced approximately 12% more offspring on Ap25 expressing leaves than on eGFP expressing leaves (20 biological replicates, χ^2^*=* 18.75, *P* < 0.001). The results indicate that Ap25 plays a role in promoting *A. pisum* feeding on *P. sativum*.

## Discussion

Here, we screened cultivars of *P. sativum* and *M. sativa* using GUS activity as a reporter and identified two *P. sativum* cultivars, ZP1130 and 1109, that are amenable to *Agrobacterium* mediated transient gene expression. We noted that *A. tumefaciens* strain AGL-1 was the most efficient strain among the three strains tested. This can be explained by the presence of extra virulent factors in this strain (Jin et al., 1987). We also noted that a few days upon infiltration with high concentration of *A. tumefaciens* (O.D._600_> 0.7), chlorosis appeared and was restricted to the agroinfiltrated area. Pruss et al. (2008) also observed that fully virulent and disarmed *A. tumefaciens* strains also triggered chlorosis restricted to the infiltrated area in tobacco plants. Although the mechanisms underlying this chlorosis have not been well understood, it could be due to a defense response to the *A. tumefaciens* involving the chloroplasts (Pruss et al., 2008).

Two tested *A. pisum* lines belonging to the pea biotype reproduced well on these two cultivars, while members of the alfalfa and clover biotypes could not survive and reproduce well on them. This indicates that these two cultivars serve as host plants of the pea biotype only and can be used to characterize candidate aphid salivary genes that may determine the compatibility of *A. pisum* biotypes with *P. sativum*. Interestingly, we found differences in aphid performances, as measured by survival and biomass, between the two *P. sativum* adapted lines on both pea cultivars. In particular, biomass production by Ar_Po_28 was about twice more than that of Ar_Po_58. Since the two lines differ in both genotype and symbiont composition (Ar_Po_28 harbors *Rickettsia* and *Serratia* secondary symbionts while Ar_Po_58 is free of any secondary symbiont, Supplementary Table S1), it is difficult to tell which factor (aphid genome or symbiont status), alone or in interaction, accounts for these differences in performances.

Although we optimized agroinfiltration in *P. sativum* to study the host specialization mechanisms in *A. pisum*, the system can be used to study the functions of *P. sativum* genes or effectors of other pea parasites. *P. sativum* is an important legume crop used in arable rotations for the production of nutritious food for both humans and animals. Various projects to identify genes involved in *P. sativum* biotic and abiotic stress resistances are ongoing (Lejeune-Henaut et al., 2008; Hamon et al., 2013; Desgroux et al., 2016) and whole-genome sequencing of *P. sativum* is underway (Alves-Carvalho et al., 2015). Therefore, the *P. sativum* research community is in need of various tools to analyze the genes of agronomical interest that will be identified in near future. Though *P. sativum* is reported to be stably transformed (Svabova et al., 2005), it remains to be a time consuming and difficult task. Recent application of virus vectors in *P. sativum* provides a new tool to express transgene in pea plant relatively quickly, but it is still time consuming (in a few weeks; Meziadi et al., 2016) and the agroinfiltration method described here provides another way to express transgenes in a few days. By using various Gateway^TM^ compatible vectors available for agroinfiltration (Karimi et al., 2002; Nakagawa et al., 2007), fusion proteins or dsRNA will be easily produced in *P. sativum* leaves. Furthermore, coexpression of a few proteins may be realized by infiltration of *A. tumefaciens* with different expression constructs.

We transiently expressed ApC002 and Ap25 in *P. sativum* leaves and examined the survival and fecundity of an *A. pisum* line of the pea adapted biotype. The aphids grew well in the clip cages fixed on the agroinfiltrated leaves and produced offspring. Since ApC002 is required for *A. pisum* feeding on *V. faba* plant, which is a universal plant of all *A. pisum* biotypes (Ferrari et al., 2008; Peccoud et al., 2009), and *in planta* (*Arabidopsis* and *N. benthamiana*) expression of MpC002 increases the fecundity of *M. persicae* feeding on the plants, we expected that ApC002 expression in *P. sativum* leaves would also increase the fecundity of the aphids. However, the survival and fecundity of the aphids fed on ApC002 expressing plants were at the same level as that of the aphids feeding on eGFP expressing plants. As C002 is one of the abundantly expressed salivary genes in *A. pisum* (Mutti et al., 2006), the aphids may produce enough of this protein and may not benefit significantly from extra production of ApC002 in *P. sativum* leaves. On the other hand, expression of Ap25 in *P. sativum* leaves increased the fecundity of the aphids. *Ap25* is an Aphididae specific gene which encodes a small protein with a signal peptide. As the protein does not show homology with known proteins, the function of Ap25 is unknown. It is possible that the protein interferes with plant defense reactions triggered by aphid feeding and facilitates nutrient acquisition from the pea plant. Carolan et al. (2011) identified more than 300 salivary genes in *A. pisum* and more than half of the identified genes encode proteins with unknown function (Carolan et al., 2011). The agroinfiltration method described here provides a mean to examine the functions of those salivary proteins in relatively short time and also allows us to investigate whether those genes are determinants of compatibility between *P. sativum* and *A. pisum* biotypes. As the second step of this study, we envisage to express salivary proteins with biotype specific sequences in the pea leaves and examine how they affect the performance of different pea biotypes installed on the leaves. Further, the agroinfiltration technique can be combined with aphid gene silencing to investigate whether a gene expressed in leaves can complement the silenced gene function (Naessens et al., 2015). Studies on plant–insect interactions at a molecular level are less advanced compared to plant-microbe interaction studies partly because it is not yet possible to transform insect herbivores. The tools to manipulate host plants, like the method described here, can provide alternative ways to examine plant–insect interactions at a molecular level and will be able to contribute to advance the field.

## Author Contributions

EG, HB, YA, CLP, KC, SM, J-CS, and AS designed the experiments. EG, HB, YA, CLP, KC, SM, KS, J-CS and AS conducted the experiments. EG, HB, GK, J-CS and AS edited the manuscript. GK, J-CS and AS provided funding for the project.

## Conflict of Interest Statement

The authors declare that the research was conducted in the absence of any commercial or financial relationships that could be construed as a potential conflict of interest.
