# Corrigendum: Optimization of Agroinfiltration in *Pisum sativum* Provides a New Tool for Studying the Salivary Protein Functions in the Pea Aphid Complex

**DOI:** 10.3389/fpls.2016.02046

**Published:** 2017-01-10

**Authors:** Endrick Guy, Hélène Boulain, Yoann Aigu, Charlotte Le Pennec, Khaoula Chawki, Stéphanie Morlière, Kristina Schädel, Grit Kunert, Jean-Christophe Simon, Akiko Sugio

**Affiliations:** ^1^INRA, UMR1349, Institute of Genetics, Environment and Plant ProtectionLe Rheu, France; ^2^Department of Biochemistry, Max Planck Institute for Chemical EcologyJena, Germany

**Keywords:** pea aphid, *Acyrtosiphon pisum*, Leguminosae, agroinfiltration, salivary proteins, biotypes, host specialization, effector

Corrigendum on Supplemental Table 2.

In the original article, description of helper plasmids in C58C1 (pGV2260) and GV3101 (pMP90) were missing. The correct information of these two strains appears below. The authors apologize for the missing information. This error does not change the scientific conclusions of the article in any way.

**Table:** 

**Bacteria**	**Features**	**References or sources**
**BACTERIA**		
*Agrobacterium tumefaciens* C58C1	Rif^r^, harbors pGV2260 (pTiB6S3ΔT-DNA)	Deblaere et al., 1985
*Agrobacterium tumefaciens* GV3101	Rif^r^, harbors pMP90 (pTiC58ΔT-DNA)	Koncz and Schell, 1986

## Conflict of interest statement

The authors declare that the research was conducted in the absence of any commercial or financial relationships that could be construed as a potential conflict of interest.
