# Supplementary material for: Optimization of Agroinfiltration in Pisum sativum Provides a New Tool for Studying the Salivary Protein Functions in the Pea Aphid Complex
Source: Front Plant Sci. 2016 Aug 9;7:1171. doi: 10.3389/fpls.2016.01171 (PMC4977312; doi:10.3389/fpls.2016.01171)
Supplement: Supplementary file 1 [file Table_1.DOCX]

**Table S1.** Aphid lineages used in this study.

| Lineage | Color | Plant origin | Location | Collection date | Secondary symbiont |
| --- | --- | --- | --- | --- | --- |
| Ar_Po_58 | Green | Pea | Ardillières, France | November 2011 | none |
| Ar_Po_28 | Green | Pea | Ardillières, France | November 2011 | *Rickettsia+Serratia symbiotica* |
| T8005 | Pink | Clover | Saint-Augustin, Canada | August 2010 | *Regiella insecticola* |
| YR2 | Pink | Clover | York, Great Britain | December 2002 | *Regiella insecticola* |
| L9Ms14 | Pink | Alfalfa | Bugey, France | August 2011 | *Hamiltonella defensa* |
