# Supplementary material for: Optimization of Agroinfiltration in Pisum sativum Provides a New Tool for Studying the Salivary Protein Functions in the Pea Aphid Complex
Source: Front Plant Sci. 2016 Aug 9;7:1171. doi: 10.3389/fpls.2016.01171 (PMC4977312; doi:10.3389/fpls.2016.01171)
Supplement: Supplementary file 2 [file Table_2.DOCX]

**Table S2.** Bacteria and plasmids used in this study.

| Bacteria and plasmids | Features | Reference or source |
| --- | --- | --- |
| Bacteria |  |  |
| *Escherichia coli* Top 10 | F- *mcrA* Δ(*mrr-hsd*RMS-*mcr*BC) Φ80*lacZ*ΔM15 Δ lacX74 *rec*A1 *ara*D139 Δ( *araleu*)7697 *gal*U *gal*K *rps*L (StrR) *end*A1 *nup*G | ThermoFisher Scientific |
|  |  |  |
| *Agrobacterium tumefaciens* C58C1 | Rif^r^, Ti plasmid cured | ([Deblaere *et al*., 1985](#_ENREF_11)) |
| *Agrobacterium tumefaciens* GV3101 | Rif^r^, Ti plasmid cured | ([Holsters *et al*., 1980](#_ENREF_21)) |
| *Agrobacterium tumefaciens* AGL-1 | Rif^r^, harbors plasmid pTiBo542ΔT-DNA | (Lazo *et al*., 1991) |
| Plasmids |  |  |
| pDONR207 | Gateway vector for entry clone construction, Gent^r^, Cm^r^ | Invitrogen |
|  |  |  |
| pENTRY-eGFP | pDONR207 derivative, eGFP ORF cloned by BP reaction, Gent^r^ | This study |
| pENTRY-GUSi | pDONR207 derivative, GUS ORF cloned by BP reaction, Gent^r^ | This study |
| pENTRY-Ap25 | pDONR207 derivative, ACYPI009919_67-435_ ORF from pea aphid line Ar_Po_58 cloned by BP reaction, Gent^r^ | This study |
|  |  |  |
| pENTRY-ApC002 | pDONR207 derivative, ACYPI008617_70-660_ ORF from pea aphid line Ar_Po_58 cloned by BP reaction, Gent^r^ | This study |
|  |  |  |
| pENTRY-ApMIF | pDONR207 derivative, ACYPI002465 ORF from pea aphid line LL01 cloned by BP reaction, Gent^r^ | This study |
| pEAQ-HT-DEST1 | Empty vector, Kan^r^, Cm^r^ | (Sainsbury, *et al*., 2009) |
| pEAQ-HT-DEST1-eGFP | p35S::eGFP Kan^r^, Cm^r^ | This study |
| pEAQ-HT-DEST1-GUSi | p35S::GUS Kan^r^, Cm^r^ | This study |
| pEAQ-HT-DEST1-Ap25 | p35S::ACYPI009919_67-435_, Kan^r^, Cm^r^ | This study |
| pEAQ-HT-DEST1-ApC002 | p35S:: ACYPI008617_70-660_, Kan^r^, Cm^r^ | This study |

**References**

Deblaere, R., Bytebier, B., De Greve, H., Deboeck, F., Schell, J., Van Montagu, M., *et al*., (1985). Efficient octopine Ti plasmid-derived vectors for *Agrobacterium*-mediated gene transfer to plants. *Nucleic Acids Res* 13, 4777-4788. doi:10.1093/nar/13.13.4777

Holsters, M., Silva, B., Van Vliet, F., Genetello, C., De Block, M., Dhaese, P., *et al.* (1980). The Functional Organization of the Nopaline *A. tumefaciens* Plasmid pTiC58. *Plasmid* 3, 212-230. doi: 10.1016/0147-619X(80)90110-9

Lazo, G.R., Stein, P.A., Ludwig R.A. (1991). A DNA transformation-competent *Arabidopsis* genomic library in *Agrobacterium*. *Nat Biotechnol* 10, 963-967. doi: 10.1038/nbt1091-963

Sainsbury, F., Thuenemann, E.C., and Lomonossoff, G.P. (2009). pEAQ: versatile expression vectors for easy and quick transient expression of heterologous proteins in plants. *Plant Biotechnol J* 7, 682-693. doi: 10.1111/j.1467-7652.2009.00434.x
