# Supplementary material for: Optimization of Agroinfiltration in Pisum sativum Provides a New Tool for Studying the Salivary Protein Functions in the Pea Aphid Complex
Source: Front Plant Sci. 2016 Aug 9;7:1171. doi: 10.3389/fpls.2016.01171 (PMC4977312; doi:10.3389/fpls.2016.01171)
Supplement: Supplementary file 3 [file Table_3.DOCX]

**Table S3.** Pea cultivars used in this study.

| Cultivars and Groups | Other names | Phylogenetic group^a^ | CRB code^b^ | Country of origin^c^ | Registration year | Reference |
| --- | --- | --- | --- | --- | --- | --- |
| ZP3508 | Champagne | II | CRB385 | FRA | - | ([Baranger *et al*., 2004](#_ENREF_3)) |
| ZP3514 | DP | II | CRB390 | - | - | ([Baranger *et al*., 2004](#_ENREF_3)) |
| ZP750 | 552 | - | CRB130 | USA | - | ([Wicker *et al*., 2003](#_ENREF_55)) |
| ZP747 | 90-2079 | - | CRB119 | USA | 1992 | (Kraft JM, 1992) |
| ZP748 | 90-2131 | - | CRB135 | USA | 1992 | ([Wicker *et al*., 2003](#_ENREF_55)), (Kraft JM, 1992) |
| ZP1109 | AeD99OSW- 50-2-5 | - | - | FRA | - | (Roux-Duparque *et al*., 2004 ; Moussart *et al*., 2007) |
| ZP793 | DSP (Dark Skin Perfection) | - | CRB134 | USA | 1995 | - |
| AP3783 | Enduro | - | - | FRA | 2007 | - |
| ZP3535 | ISARD | - | - | FRA | 2005 | - |
| ZP1130 | PI180693 | - | CRB128 | DEU | - | ([Wicker *et al*., 2003](#_ENREF_55)) |
| ZP690 | PUGET | VI sgIII | CRB412 | GBR | - | ([Baranger *et al*., 2004](#_ENREF_3)) |
| ZP1124 | JI296 | VI sgIII | CRB448 | FRA | - | ([Baranger *et al*., 2004](#_ENREF_3)) |
| AP3830 | FP (CE101) | VII | CRB383 | FRA | - | ([Baranger *et al*., 2004](#_ENREF_3)) |
| ZP3495 | BACCARA | VIII sgI | CRB423 | FRA | 1991 | ([Wicker *et al*., 2003](#_ENREF_55)) |
| WP1018 | EIFFEL | VIII sgI | - | FRA | 1993 | ([Baranger *et al*., 2004](#_ENREF_3)) |
| ZP3664 | SOLARA | VIII sgII | - | HOL | 1986 | ([Baranger *et al*., 2004](#_ENREF_3)) |
| ZP3570 | TERESE | VIII sgII | CRB413 | DEN | 1988 | ([Baranger *et al*., 2004](#_ENREF_3)) |
|  |  |  |  |  |  |  |

a: as described ([Baranger *et al*., 2004](#_ENREF_3)), sg (sub-group); b: Line codes in the Biological resource center (CRB) database of INRA Dijon, France; c: Country codes ISO3166-1, FRA (France), USA (United states of America), DEU (Germany), GBR (Great Britain), Hol (Holland), DEN (Denmark).

**References**

Baranger, A., Aubert, G., Arnau, G., Laine, A.L., Deniot, G., Potier, J., *et al*., (2004). Genetic diversity within *Pisum sativum* using protein- and PCR-based markers. TAG. Theoretical and applied genetics. *Theoretische und angewandte Genetik* 108, 1309-1321. doi: 10.1007/s00122-003-1540-5

Kraft JM. Registration of 90-2079, 90-2131, and 90-2322 pea germplasms (1992). Crop Sci. *32*, 1076. doi: 10.2135/cropsci1992.0011183X0032000

40063x

Moussart A., Devaux C., Muel F., Pilet-Nayel M., Baranger A., Tivoli B. *et al*., (2007). Improving partial resistance to *Aphanomyces* root rot in GSP breeding program. 3^rd^ International *Aphanomyces* Workshop on Legumes, France.

Roux-Duparque M., Boitel C., (2004). Breeding peas for resistance to *Aphanomyces* root rot: current main outputs of three breeding programes. Eur Conf Grain Legumes, France.

Wicker, E., Moussart, A., Duparque, M., and Rouxel, F. (2003). Further contributions to the development of a differential set of pea cultivars (*Pisum sativum*) to investigate the virulence of isolates of *Aphanomyces euteiches*. *Eur J Plant Pathol* 109, 47-60. doi: 10.1023/A:1022020312157
