# Supplementary material for: Optimization of Agroinfiltration in Pisum sativum Provides a New Tool for Studying the Salivary Protein Functions in the Pea Aphid Complex
Source: Front Plant Sci. 2016 Aug 9;7:1171. doi: 10.3389/fpls.2016.01171 (PMC4977312; doi:10.3389/fpls.2016.01171)
Supplement: Supplementary file 4 [file Table_4.DOCX]

**Table S4.** Primers used in this study.

| Name | Sequence (5'->3')^a^ |
| --- | --- |
| ATTB1 | ACAAGTTTGTACAAAAAAGCAGGC |
| ATTB2 | ACCACTTTGTACAAGAAAGCTGGG |
| GFP-Fw | AAAAAGCAGGCTCC**ACC**ATGGTGAGCAAGGGCGAGGAGCTG |
| GFP-Rv | AGAAAGCTGGGTGTTACTTGTACAGCTCGTCCATGCCGAG |
| GUS-Fw | AAAAAGCAGGCTCC**ACC**ATGTTACGTCCTGTAGAAACC |
| GUS-Rv | AGAAAGCTGGGTGTCATTGTTTGCCTCCCTGCTG |
| AP25-Fw | AAAAAGCAGGCTCC**ACC**ATGACAAAAACTGAAAAATCTGAC |
| AP25-Rv | AGAAAGCTGGGTTTATTTTTTGTTTCATTTTT |
| APC002-Fw | AAAAAGCAGGCTCC**ACC**ATGGATTGGTCTGCCGCTGAACC |
| APC002-Rv | AGAAAGCTGGGTTTAAAAACGTCGAAG |

a: underlined and bold sequences indicate *att* sites for cloning purposes and Kozak consensus, respectively.
